# Mode of Arresting Hemorrhage from Extraction of a Tooth

**Published:** 1846-10

**Authors:** 


19. *Mode of arresting Hemorrhage from extraction of a tooth.*—The editor of the *Lond. Med. Gaz.* states that he has known the most obstinate bleeding, following the extraction of a tooth and continuing some hours, arrested by the use of the oil of turpentine on a pledget of lint, kept over the bleeding surface for a short time by moderate pressure.—*Med. News*.

